# Enteric Nervous System Regulation of Intestinal Stem Cell Differentiation and Epithelial Monolayer Function

**DOI:** 10.1038/s41598-018-24768-3

**Published:** 2018-04-20

**Authors:** Marissa Puzan, Sanjin Hosic, Caroline Ghio, Abigail Koppes

**Affiliations:** 1 Department of Chemical Engineering, Northeastern University, Boston, MA 02115 USA; 2 Department of Biology, Northeastern University, Boston, MA 02115 USA

## Abstract

The Enteric Nervous System (ENS) is a complex network of neurons and glia, which regulates sensorimotor function throughout the gastroinestinal tract (GI). Here we investigated the role of the ENS and intestinal myofibroblasts in the maintenance of a primary intestinal epithelial barrier through regulation of monolayer permeability, cytokine production, and differentiation of intestinal stem cells. Utilizing a novel, *in vitro*, transwell-based coculture system, murine small intestinal stem cells were isolated and cultured with ENS neurons and glia or subepithelial myofibroblasts. Results show that the ENS contributes to regulation of intestinal stem cell fate, promoting differentiation into chemosensory enteroendocrine cells, with 0.9% of cells expressing chromogranin A when cultured with ENS versus 0.6% in cocultures with myofibroblasts and 0.3% in epithelial cultures alone. Additionally, enteric neurons and myofibroblasts differentially release cytokines Macrophage Inflammatory Protein 2 (MIP-2), Transforming Growth Factor beta 1 (TGF-β1), and Interleukin 10 (IL-10) when cultured with intestinal epithelial cells, with a 1.5 fold increase of IL-10 and a 3 fold increase in MIP-2 in ENS cocultures compared to coculture with myofibroblasts. These results indicate the importance of enteric populations in the regulation of intestinal barrier function.

## Introduction

The intestinal epithelial cell barrier plays a vital role in gut homeostasis through its regulation of the flux of nutrients, toxins, and bacteria into the body from the intestinal lumen^1^. Dysregulation of this barrier, often referred to as “leaky gut”, leads to intestinal or systemic inflammation and intestinal illnesses^2^, such as inflammatory bowel disease (IBD), Crohn’s Disease, and colitis, which affect more than a million people in the United States each year^3^. Proper function of the epithelial barrier depends on a variety of factors, including differentiation of intestinal stem cells into desired epithelial phenotypes, including absorptive enterocytes, mucus secreting goblet cells, secretory enteroendocrine cells, and antimicrobial paneth cells, among others^4–6^, but function also depends on the regulation of epithelial permeability^7,8^. Epithelial permeability is largely regulated by the expression of tight junction associated proteins, such as zona occludens (ZO), which effectively connects cells, sealing the extracellular space between them and preventing the undesired transport into the body. Both stem cell differentiation and ZO protein expression depend on cues from cells surrounding the intestinal niche. These cells, including enteric neurons and glia, may provide a target for the treatment of intestinal inflammatory diseases like IBD, through regulation of barrier function or immune responses^9–13^. However, the inclusion of these trophic cells that may influence the epithelial barrier are lacking in current tissue engineered models.

Below the intestinal epithelial layer lies a vast network of neurons and glia, called the enteric nervous system (ENS). The ENS is responsible for the regulation of all sensory and motor function within the GI, which it can perform independently of the brain, thus it is frequently termed “the brain in the gut.” Recent investigations have implicated the role of ENS, particularly enteric glia, and subepithelial myofibroblasts (ISEMF)^14^, in the regulation of the intestinal epithelial barrier through cytokine production and regulation of the expression of tight junction associated proteins,^9,11,15–17^. In murine models, ablation of glia has also been reported to induce inflammation and intestinal disturbances, indicating the importance of glial participation in barrier function^9,12^. However, to date, no model has incorporated a primary epithelial monolayer with enteric populations to parse the specific roles of ENS and epithelial communication in barrier regulation.

In the small intestine, enteric glia reside around crypts and throughout intestinal villi, while enteric neurons lie below intestinal crypts within the submucosa as well as between longitudinal and circular smooth muscle layers^18^. Projections from both enteric neurons and glia directly contact the epithelium through junctions with enteroendocrine sensory cells residing in the epithelium^19,20^, and neurons and glia release a variety of signaling molecules, including neurotransmitters and cytokines. Thus there may be extensive bidirectional communication between the epithelium and ENS, with the neuroendocrine pathways playing a role in maintenance of intestinal epithelial barrier integrity and contributing to intestinal diseases^21^. Intestinal inflammation is accompanied by alterations in neurochemical coding of enteric neurons in irritable bowel syndrome^22–24^; however, the specific soluble or contact dependent factors produced by the ENS to impact epithelial barrier function and intestinal stem cell fate remain unknown, as they are difficult to parse in the complex *in vivo* environment.

Because of its close proximity to the small intestinal epithelium, we hypothesize that the ENS plays a critical role in the regulation of the intestinal stem cell niche and epithelial barrier function both through direct cell-cell connections and through the release of trophic factors. As a first step, here we investigated the impact of enteric produced soluble factors on intestinal stem cell differentiation and barrier permeability in a controlled, *in vitro* platform. The use of intestinal stem cells not only provides a more ‘healthy’ epithelial layer as opposed to traditionally used epithelial cancer cell lines, but it allows exploration into the regulation of stem cell differentiation by these trophic cells. In addition to ENS contribution, the impact of intestinal myofibroblasts on stem cell fate and epithelial health was assessed. This model enables controlled investigation of the cross talk between the epithelium and enteric neurons and glia, and allows future studies on the impact of various intestinal metabolites or bacteria on overall epithelial and neural health.

## Results

### Overview of the Development of Coculture Model

**Figure 1:**
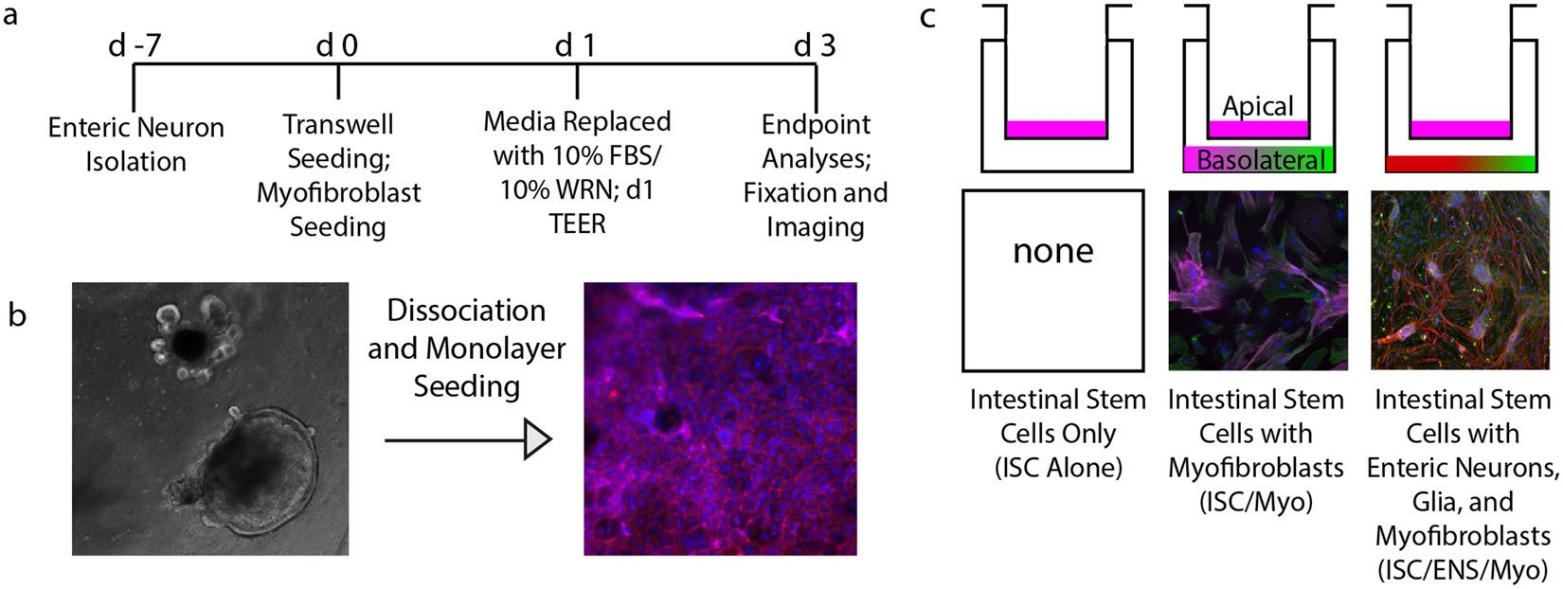
Experimental Timeline and Setup. (**a**) Primary enteric neurons were harvested 7 days prior to the start of coculture and monolayer seeding (day 0). Monolayers were grown in stem cell media for 24 hours, which was replaced with differentiation media. (**b**) Murine duodenal intestinal stem cells (ISC) were harvested and grown as organoids prior to dissociation and monolayer seeding: (**c**) either alone (ISC alone), above subepithelial myofibroblasts (ISC/Myo), or above complete enteric cultures, containing enteric neurons, enteric glia, and myofibroblasts (ISC/ENS/Myo).

The coculture system described herein was developed to determine interactions between primary intestinal epithelial cells and primary enteric neurons and glia. With that in mind, duodenal LGR5+ intestinal stem cells were isolated^5,25,26^ and differentiated into primary epithelial monolayers, as these multipotent cells can become one of the various epithelial phenotypes found *in vivo*, Fig. 1. Epithelial monolayers were grown either alone, with enteric neurons, glia, and intestinal myofibroblasts, or with only intestinal myofibroblasts.

### Characterization of Enteric Cultures

**Figure 2:**
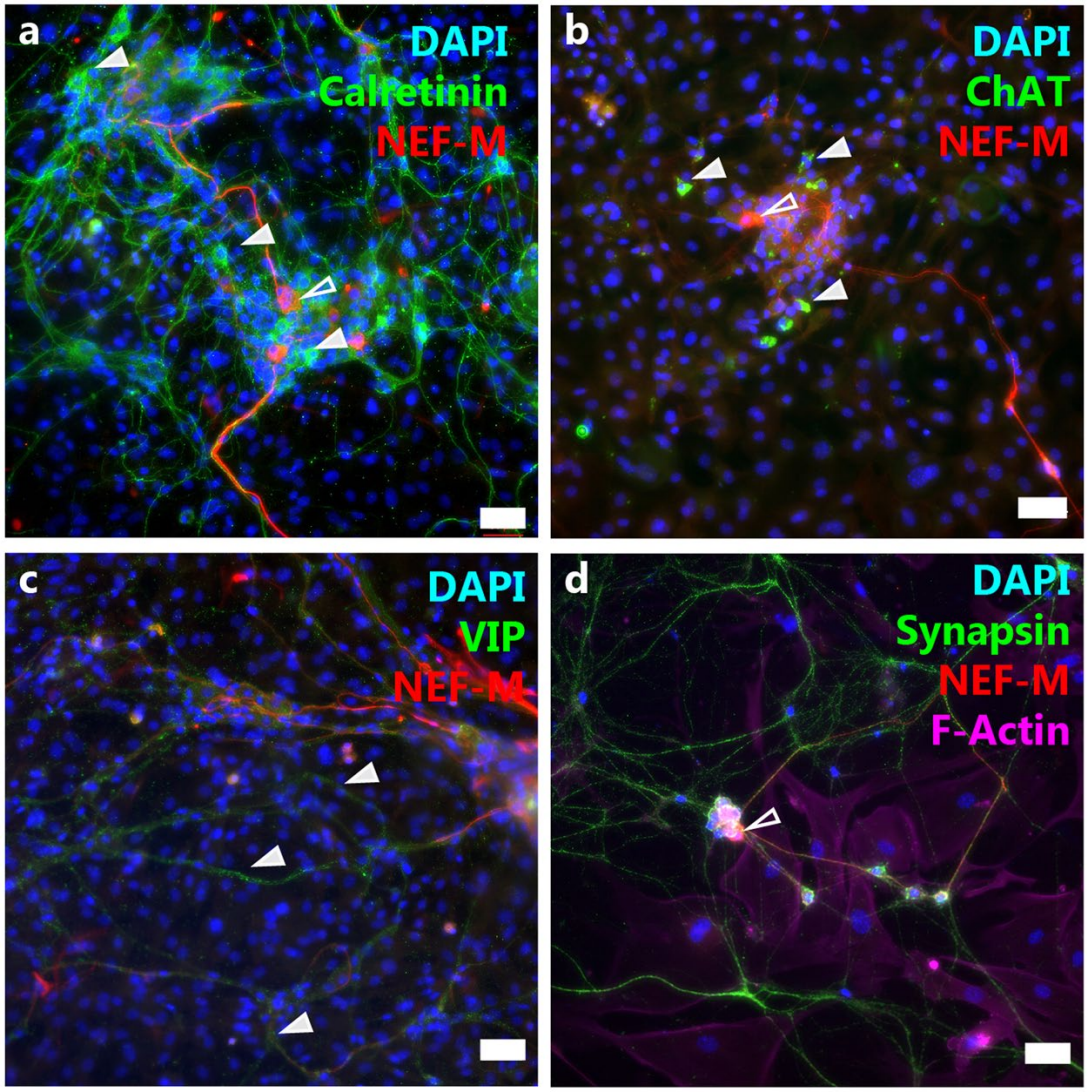
Characterization of Cultured Enteric Neurons. Cultures of enteric neurons show reactivity to 160 kd neurofilament (indicated by open arrows), calretinin (**a**), choline acetyletransferase (**b**), (cell bodies indicated with arrows), and VIP (**c**), (VIPergic fibers indicated by arrows). (**d**) Synapsin highlights all neural fibers and F-Actin reactivity shows myofibroblasts. Scale: 50 μm.

**Figure 3:**
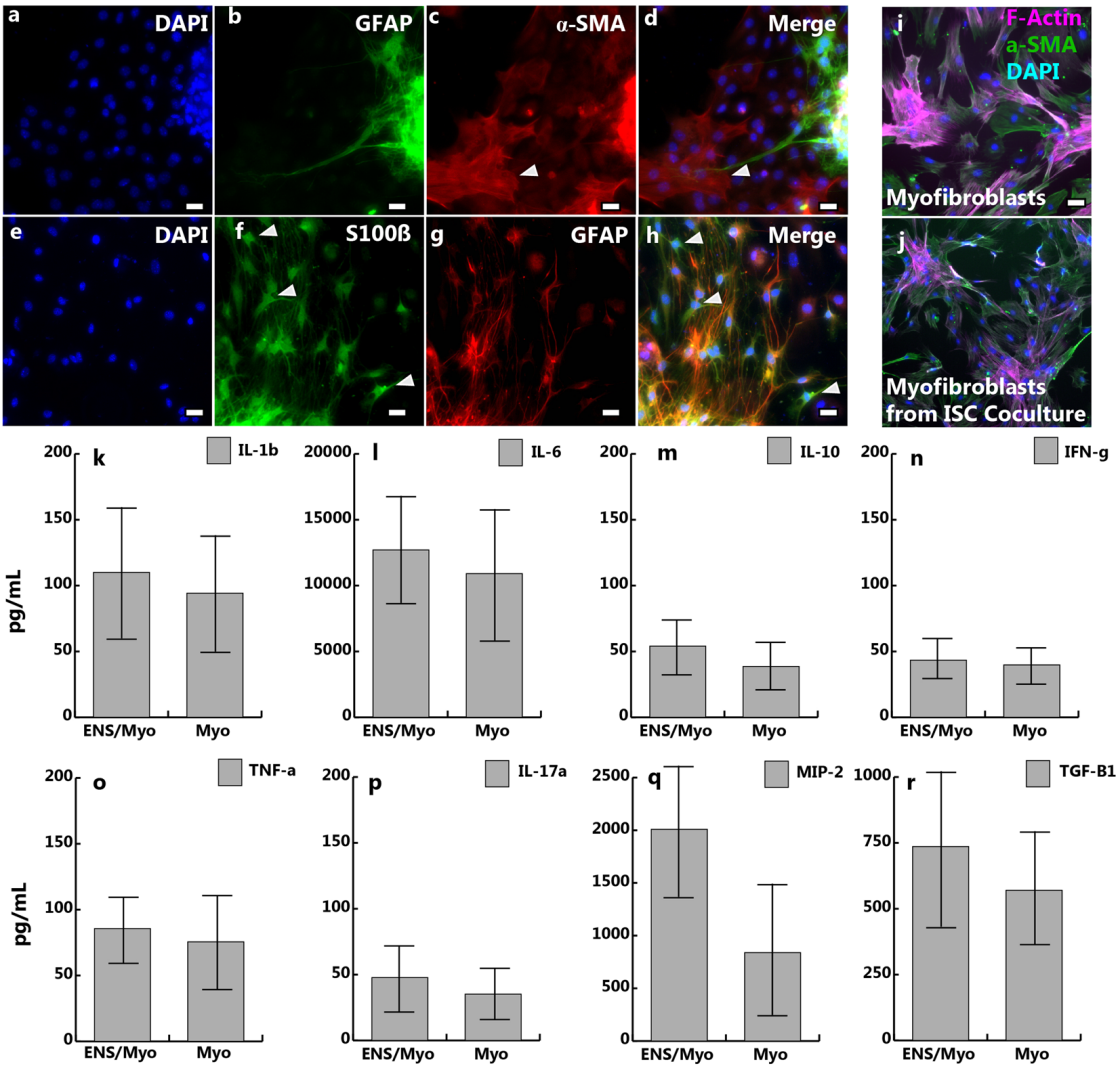
Non-neuronal Characterization of Subepithelial Cultures. (**a**–**d**) In addition to neurons, ENS derived cultures contain both enteric glia (GFAP and S100β) and myofibroblasts (α-SMA), which are indicated with arrows. (**e**–**h**) Enteric glia express S100β (**f**), with some coexpressing GFAP (**g**). Glia expressing only S100β are indicated with arrows. (**i**,**j**) Cultures of intestinal derived myofibroblasts broadly express α-smooth muscle actin (α-SMA), in addition to F-actin, and visually appear altered after coculture with intestinal monolayers, with a more slender morphology. (**k**–**r**) Basal levels of cytokine expression after 48 hours of culture for myofibroblasts and complete ENS cultures in a basal state without the addition of intestinal monolayers. No significant differences are seen between myofibroblast cultures and complete ENS cultures, which contain neurons, glia, and myofibroblasts. Error bars: SD. Scale Bars: (**a**–**d**) 20 μm, (**i**,**j**) 50 μm.

Immunofluorescent imaging was used to characterize both ENS and myofibroblast cultures. Neurons in culture express 160 kd neurofilament (NEF-M), calretinin, choline acetyltransferase (ChAt), and vasoactive intestinal peptide (VIP), as seen in Fig. 2. Immunoreactivity to serotonin and tyrosine hydroxylase were not observed (images not shown). Further characterization showed the presence of non-neuronal cell types, including enteric glia and intestinal myofibroblasts in ENS cultures. Figure 3(a–h) shows expression of glial fibrillatory acid protein (GFAP) and S100β, both indicative of enteric glia, α smooth muscle actin (α-SMA) for myofibroblasts, and DAPI for cell nuclei in complete enteric derived cultures. Although there are large populations of neurons and glia, myofibroblasts are also apparent. Myofibroblast cultures contain large proportions of cells expressing α-SMA (i, j). Interestingly, morphological alterations are apparent in myofibroblast cultures after their coculture with intestinal monolayers compared to their culture alone. Myofibroblasts from coculture experiments exhibit a more elongated morphology than when cultured alone, Fig. 3(i,j).

Basal multiplex cytokine expression of interleukins (IL) IL-1β, IL-6, IL-10, IL-17a, interferon gamma (IFN-γ), tumor necrosis factor alpha (TNF-α), Macrophage inflammatory protein 2 (MIP-2), and transforming growth factor 1 beta (TGF-β1) were not significantly different between myofibroblast cultures and complete enteric cultures without the addition of the epithelium (Fig. 3. Levels of cytokines were as follows for ENS cultures: IL-1β: 110.0 +/− 71.3 pg/mL, IL-6: 1.27 e4 +/− 6.46 e3 pg/mL, IL-10: 54.0 +/− 27.9 pg/mL, IFN-γ: 43.4 +/− 20.6 pg/mL, TNF-α: 85.6 +/− 30.1 pg/mL, IL-17a: 47.6 +/− 35.0 pg/mL, MIP-2: 2009 +/− 272 pg/mL, and TGF-β1: 763 +/− 212 pg/mL. Cytokine expression in myofibroblast cultures were as follows: IL-β1: 94.2 +/− 68.4 pg/mL, IL-6: 1.10 e4 +/− 7.14 e3 pg/mL, IL-10: 38.6 +/− 26.6 pg/mL, IFN-γ: 39.7 +/− 24.0 pg/mL, TNF-α: 75.6 +/− 44.7 pg/mL, IL-17a: 35.2 +/− 27.2 pg/mL, MIP-2: 839 +/− 921 pg/mL, and TGF-β1: 194 +/− 325 pg/mL. MIP-2 and TGF-β1 differed the most between the two cultures, although differences were insignificant.

### Enteric Cells and Myofibroblasts Decrease Monolayer Permeability

**Figure 4:**
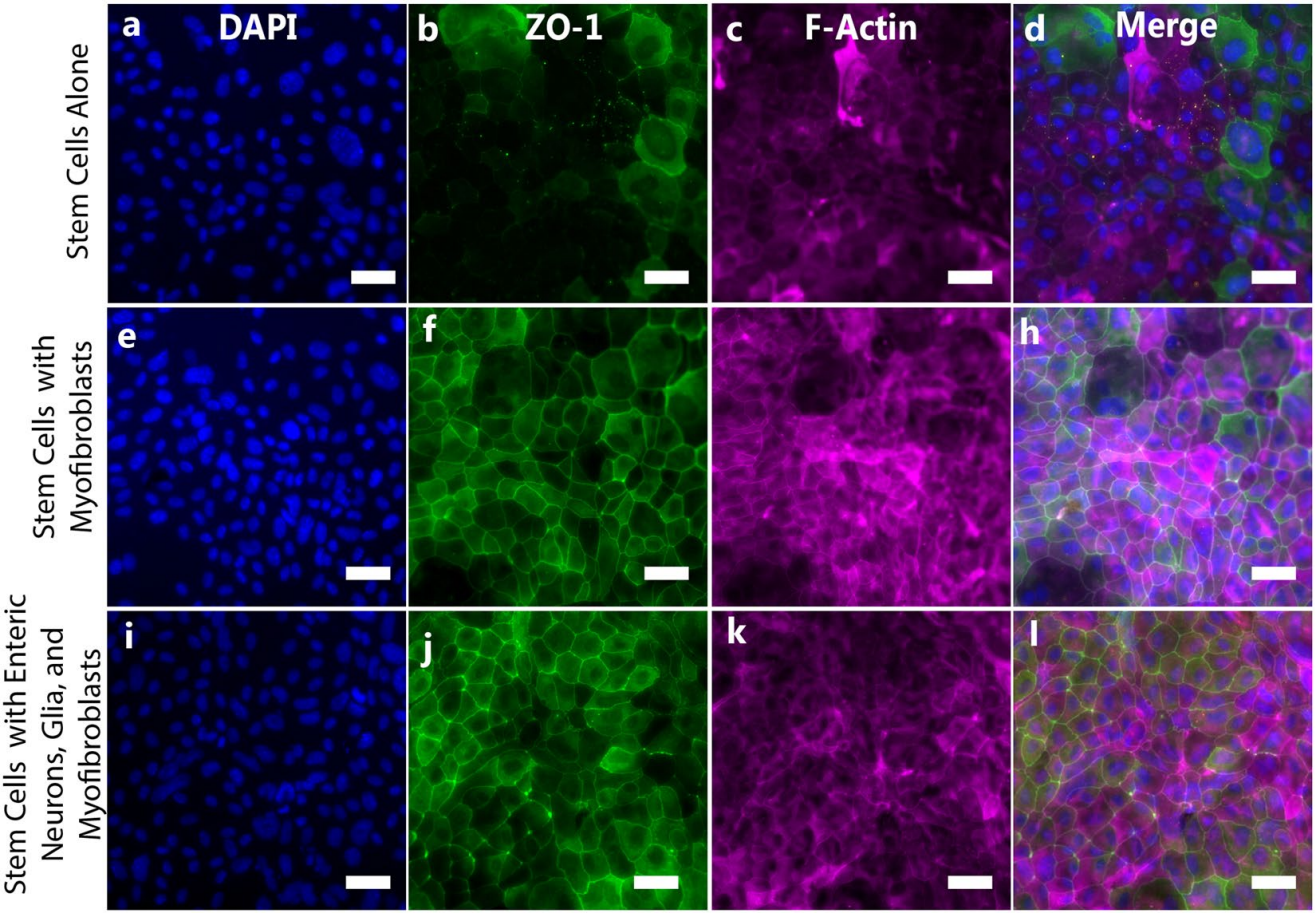
Epithelial Monolayer Morphologies and Structural Protein Expression are Altered in the Presence of Subepithelial Cells. Subepithelial myofibroblasts and enteric neurons and glia increase expression of both ZO-1 (**b**,**f**,**j**) and F-Actin (**c**,**g**,**k**) in intestinal stem cell derived epithelial monolayers, likely decreasing permeability and increasing overall monolayer integrity. Scale: 25 μm.

Because epithelial health is often correlated with low permeability through the epithelial barrier, overall integrity of the stem cell derived epithelial monolayer for each coculture system was compared via expression of tight junction associate protein, zona occludens 1 (ZO-1), transepithelial resistance across the monolayer, and apical to basolateral dextran permeability. Immunofluorescent images of monolayers, Fig. 4, show increased expression of ZO-1 and F-Actin in cocultures compared to epithelial monolayers alone. F-actin expression appears slightly higher in myofibroblast cocultures compared to that of complete enteric cocultures (Fig. 4(g,k)), while ZO-1 appears similar across both cocultures (Fig. 4(f,j)).

**Figure 5:**
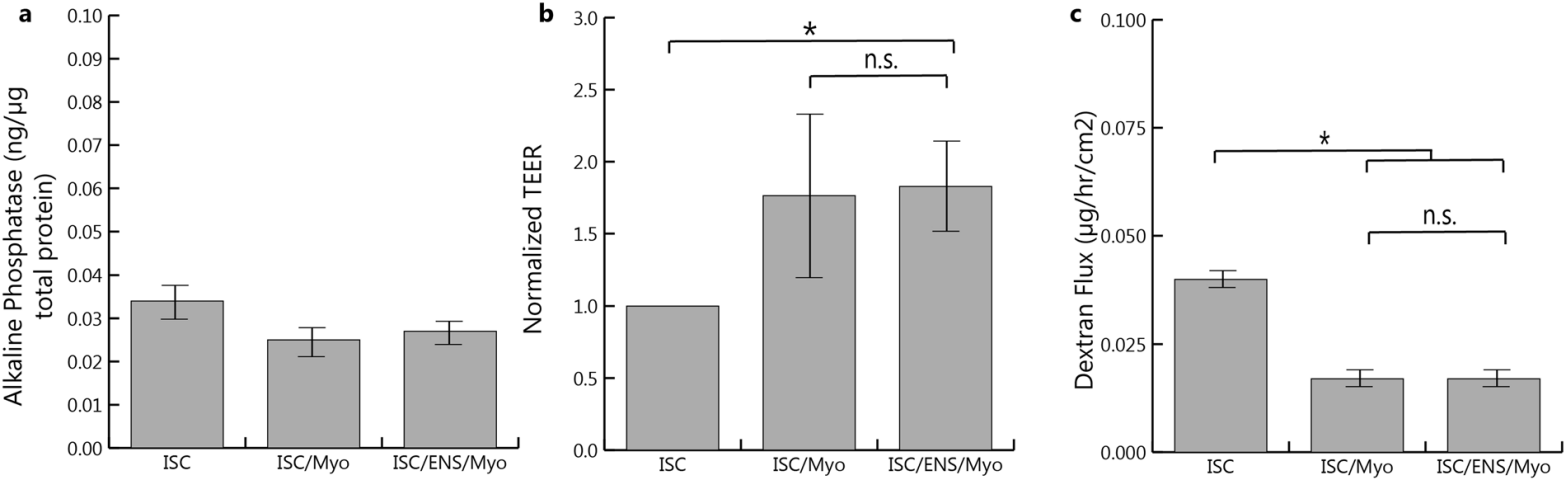
Analysis of Monolayer Function and Integrity (**a**) Epithelial expression of alkaline phosphatase, a brush border enzyme, is not affected by coculture with myofibroblasts or enteric neurons and glia. (**b**) Transepithelial resistance (TEER) increases almost twofold compared to epithelium alone when either myofibroblasts or complete enteric cultures are present. (**c**) Flux of FITC-coordinated dextran from the apical to basolateral side of the transwell is significantly reduced by the presence of myofibroblasts (p = 0.05) or ENS (p = 0.03), (*), indicating decreased permeability. Error bars: SD.

Monolayer health was also assessed via expression of alkaline phosphatase, a brush border enzyme found on epithelial cells, which helps to break down cholesterol and fatty acids^27^. No significant changes in alkaline phosphatase were observed (Fig. 5(a)), with average activities of 0.088 +/− 0.032 ng/μg total protein for epithelium alone, 0.080 +/− 0.025 ng/μg total protein for epithelium cocultured with myofibroblasts, and 0.061 +/− 0.015 ng/μg total protein for epithelium cocultured with enteric neurons, glia, and myofibroblasts. Transepithelial electrical resistance (TEER), an indication of barrier ion permeability, for myofibroblast coculture was 1.76× that of the epithelium alone (p = 0.04), while coculture with ENS and myofibroblasts was 1.82× that of the epithelium alone (p = 0.005), Fig. 5(b). Flux of FITC-dextran through the epithelial monolayer was 0.04 +/− 0.008 μg per hour per cm^2^, while coculture with myofibroblasts was 0.017 +/− 0.011 μg per hour per cm^2^ (p = 0.049) and with ENS was 0.017 +/− 0.009 μg per hour per cm^2^ (p = 0.032), Fig. 5(c). There was no significant difference between TEER values or dextran flux for the two different subepithelial cocultures with the epithelium.

### Monolayer Differentiation is Altered in the Presence of ENS or Myofibroblast Cultures

**Figure 6:**
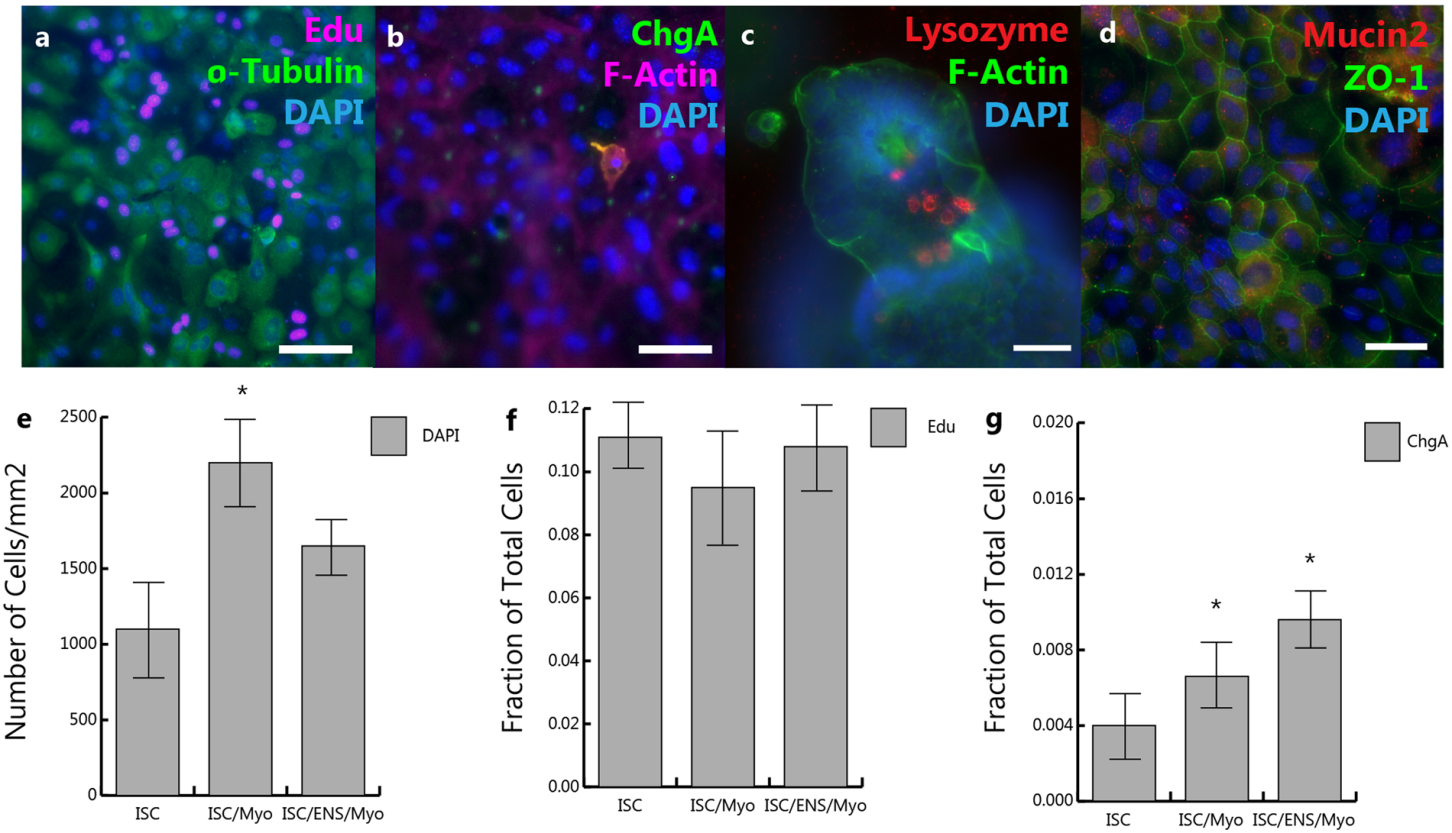
Proliferation and Differentiation in Epithelial Monolayers (**a**),(**f**), Upon fixation and analysis at day 3, epithelial monolayers maintain some proliferative ability, as determined by Edu incorporation, which was similar across all conditions. (**b**) Enteroendocrine cells in monolayers express Chromogranin A (ChgA). (**c**) Lysozyme, indicative of paneth cells, was expressed in 3D organoids, but not in differentiated monolayers. (**d**) Muc2 expression in indicates the presence of goblet cells in the epithelium. (**e**) Monolayers cultured with myofibroblasts were more dense (based on nuclei density) than monolayer only (*) p = 0.018, a 100% increase over epithelial only cultures and 40% increase over ENS cocultures. (**f**) There was no change in Edu incorporation, indicating proliferating cells. (**g**) Both myofibroblasts and ENS derived cultures increase differentiation of intestinal stem cells into enteroendocrine cells, myofibroblasts p = 0.08, ENS p = 0.003, compared to the epithelium alone (*). There was no difference in expression between myofibroblast and ENS cultures. Scale Bars: 50 μm.

In this *in vitro* system, it was observed that the presence of trophic cells altered the differentiation profile of the intestinal stem cell derived epithelial monolayers. In immunofluorescent images, it was apparent that both ENS cultures and myofibroblast cultures seem to regulate cell density in epithelial monolayers. At day 3, myofibroblast coculture produced monolayers with significantly more cells per mm^2^, 2300 +/− 435 cells per mm^2^, compared to the epithelium alone, 1100 +/− 280 cells per mm^2^ (p = 0.018) and cocultures with ENS, 1650 +/− 420 cells per mm^2^. This was not due to proliferation, as Edu incorporation from day 2 to day 3 was similar for all conditions, with roughly 10% of cells maintaining proliferative ability, Fig. 6(f). Within monolayers, cells positive for Mucin2 and ChgA, indicative of goblet and enteroendocrine cells, were observed. No lysozyme expression was observed in monolayers, although it was observed in 3D organoids prior to dissociation and seeding, Fig. 6(c). Finally, the fraction of cells expressing ChgA was significantly increased in cocultures with myofibroblasts 0.006+/−0.004 versus the epithelium alone 0.004 +/− 0.004, p = 0.08, and with ENS, 0.009 +/− 0.004, p = 0.003 versus epithelial only cultures.

### Cytokine Production by the ENS and Signaling with the Stem-Cell Derived Epithelium

Apical and basolateral transwell chambers were sampled to determine cytokine production by both epithelium and subepithelial cells. As noted earlier (Fig. 3), both myofibroblasts and ENS co-cultures produce a variety of cytokines, including IL-1β, IL-6, IL-10, IFN-γ, TNF-α, IL-17a, MIP-2, and TGF-β1, all of which have various roles in the regulation of intestinal inflammation. Epithelial cells also produced low quantities of IFN-γ (apical secretion: 13.7 pg/mL +/− 10.4 pg/mL, basolateral secretion: 6.4 pg/mL +/− 4.0 pg/mL), TNF-α (apical secretion: 20.3 pg/mL +/− 16.8 pg/mL, basolateral secretion: 6.9 pg/mL +/− 5.9 pg/mL), and TGF-β1 (apical secretion: 334.4 pg/mL +/− 40.9 pg/mL, basolateral secretion: 548.8 pg/mL +/− 208.3 pg/mL). Although there were no significant differences in cytokine production between monocultures of myofibroblasts or complete ENS, Fig. 3(k–r), the addition of epithelium containing transwells to these cultures stimulated production of both pro- and anti- inflammatory cytokines. Levels of IL-10 and TGF-β1 were increased in ENS cocultures compared to basal levels in ENS controls: IL-10, (70.4 pg/mL vs. 54.0 pg/mL), p = 0.085; TGF-β1, (1584 pg/mL vs. 763.2 pg/mL), p = 0.083, suggesting bidirectional signaling between the ENS and epithelium. Finally, levels of IL-10 (70.4 pg/mL +/− 46.2 pg/mL in ENS vs. 28.1 pg/mL +/− 19.2 pg/mL in myofibroblasts, p = 0.04), MIP-2 (2139 pg/mL +/− 330.0 pg/mL in ENS vs. 504 pg/mL +/− 532 pg/mL in myofibroblasts, p = 0.01), and TGF-β1 (1584 pg/mL +/− 288 pg/mL in ENS vs. 748 pg/mL +/− 153 pg/mL in myofibroblasts, p = 0.02) were increased on the basolateral side of the transwells in complete ENS cocultures compared to myofibroblast only cocultures, thus may be produced by enteric neurons or glia at a higher rate than by myofibroblasts.

**Figure 7:**
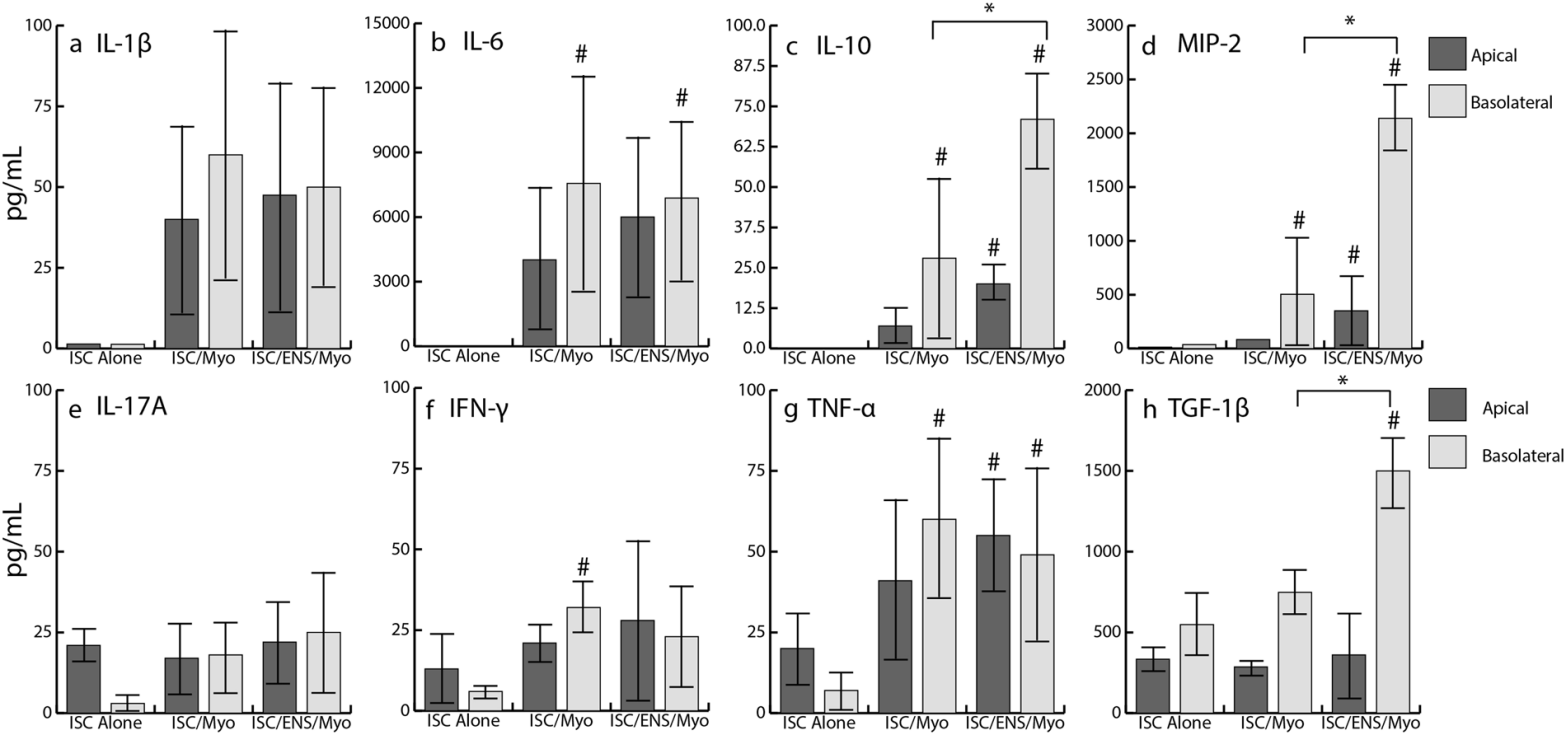
Cytokine Expression within Intestinal Epithelial Cocultures. Both Myofibroblasts and ENS derived cultures produced a variety of cytokines that interact with the epithelial barrier. (**a**–**c**) Production of (**a**) IL-β1, (**b**) IL-6, and (**c**) IL-10 were increased in both basolateral and apical sides of transwells in cocultures compared to intestinal stem cell (ISC) derived monolayers. (Significance of p < 0.05 indicated by #) (**c**) Myofibroblasts and ENS cultures produced IL-10, which were significantly above basolateral levels in control cultures (Myofibroblasts: p = 0.04, ENS: p = 1.8 e-6, (#)). Additionally, basolateral levels in ENS cultures were higher than in myofibroblast cultures, p = 0.04 (*), and apical levels in ENS cultures were higher than epithelium alone (#), p = 0.01). (**d**) MIP-2 followed a similar trend, with both basolateral chambers above control levels, (#) p = 0.01 and p = 0.0003, with ENS also significantly higher than myofibroblast levels, (*), p = 0.01. (**e**) IL-17A showed no changes. (**f**) IFN-γ and (**g**) p = 0.10. (**g**) TNF-α were increased in basolateral myofibroblast chambers, (#), p = 0.10 and p = 0.04. TNF-α was also increased in apical and basolateral ENS coculture chambers, (#), p = 0.1 and p = 0.07, respectively. (h) TGF-β1 was increased in ENS coculture basolateral compartments, (#), p = 0.02, and ENS levels were higher than those produced by myofibroblasts, (*), p = 0.02. Most notably, MIP-2, IL-10, and TGF-β1 expression significantly increased with the addition of ENS cultures compared to cocultures with myofibroblasts (*).

As epithelial cells themselves produce few cytokines, the addition of trophic cells led to increases compared to basal levels in the cultures of epithelial monolayers alone, 7 (a–h). There was little to no detection of IL-1β, IL-6, IL-10, and MIP-2 in cultures of only epithelial cells. There were no increases in production of IL-17A when either trophic cell cultures were added, Fig. 7(e). IFN-γ was increased by 1.4 fold in the basolateral chambers of cocultures with myofibroblasts, Fig. 7(f), p = 0.1. TNF-α increased by 7.8 fold on the basolateral side of myofibroblast cocultures (p = 0.04) and increased 1.7 fold in the apical side (p = 0.1) and 6 fold in the basolateral side (p = 0.08) of cocultures with enteric cultures, Fig. 7(g). The only significant increase in levels of TGF-β1 occurred on the basolateral side of ENS cocultures, with a 1.9 fold increase compared to basolateral levels in epithelial cultures, Fig. 7(h), p = 0.02.

When levels of cytokines on the apical versus basolateral of the simulated epithelial barrier within this model were compared, a few cytokines levels were altered. In epithelial only cultures, there was a significant, 6 fold increase in apical levels of IL-17A, p = 0.02. In cocultures with myofibroblasts, levels of TGF-β1 were different, with basolateral levels 1.6 fold higher than apical levels, p = 0.008. Finally, in ENS cocultures, levels of IL-10, MIP-2, and TGFβ1 were all higher in basolateral chambers, likely because of their production by the ENS. IL-10 was 2.3 fold higher in the basolateral chamber, p = 0.01. MIP-2 was 5 fold higher, p = 0.002. TGF-β1 was 6.3 fold higher in the basolateral chamber, p = 0.03.

## Discussion

The recent development of a Matrigel^®^ based 3D culture system for the expansion of intestinal stem cells^28^ and their subsequent differentiation^5^ has advanced our understanding of the intestinal microenvironment, epithelial health, and intestinal inflammation. Traditional models of the intestinal epithelium relied on epithelial cancer cell lines, such as colorectal adenocarcinoma derived Caco-2 or HT29 cells, which provide incomplete representations of a healthy intestinal epithelium. These lines have limited ability to represent the varied epithelial phenotypes, each of which serves a specific purpose in regards to intestinal health and barrier integrity^29^. Recent studies have utilized primary LGR5+ intestinal stem cells to investigate the effects of various other cell types, including macrophages^30^, myofibroblasts^14^, and bacteria, on intestinal function^31^. However, many other cellular components, including enteric neurons and glia, have not been explored in these systems. Here we developed and characterized an *in vitro*, transwell based model of the neural-epithelial microenvironment, which we envision will allow for the addition of metabolites or microbes to apical or basolateral compartments and subsequent study of their effects on both intestinal and neural health.

To date, few models have incorporated enteric neurons into cultures of stem cell derived intestinal epithelial cells. A recent study showed pluripotent stem cell derived 3D human intestinal organoids (HIOs) cocultured with vagal neural crest cells led to the innervation of HIOs^32,33^. These neural cells differentiate and express both β-III tubulin and s100β. HIOs with crest derived neurons elicited alterations in epithelial differentiation, as determined through broad changes in gene expression relating to various epithelial phenotypes^32,33^. For HIOs cultured with neurons, there were decreases in expression of enterocyte and goblet cell markers, differential alterations in expression of markers for both paneth and enteroendocrine cells, with decreases in expression of both CHGA and lysozyme, demonstrating the involvement of the ENS on epithelial cell fate. Furthermore, cultures with a neural component displayed decreased MUC2 expression and increased proliferation. Although informative for determining the influence of the ENS on intestinal stem cell differentiation, this model requires implantation of organoids, thus systematic perturbations of this system are invasive and impractical; a highly controllable *in vitro* model is necessary for future studies with higher throughput of epithelial-ENS communication.

Because the intestinal microenvironment is complex, with a variety of cues from surrounding cells in the epithelium, the lamina propria, the contents of intestinal lumen, and resident microbiota, recapitulating the intestinal stem cell niche *in vitro* is difficult. *In vivo*, intestinal stem cells lie at the base of intestinal crypts and rely on Wnt and Notch signaling from surrounding cells to maintain their proliferative ability; thus, these molecules are necessary for *in vitro* stem cell culture. Levels of Wnt and Notch further influence differentiation into various cell types, including enterocytes, goblet cells, enteroendocrine cells, and paneth cells, which are necessary for proper function of the intestinal cell barrier^34^. *In vitro*, these molecules drive differentiation toward specific phenotypes, and their removal or inhibition is often required to obtain varied epithelial differentiation^35^. Because cells such as myofibroblasts, neurons, and glia, among others, secrete factors such as Wnt, Notch^34^, and BMP4^36^ to promote differentiation of stem cells and regulate barrier permeability, their addition to *in vitro* cultures may provide a more accurate model of the intestinal epithelial barrier.

Although the ENS has been implicated in intestinal diseases, its role in regulation of the epithelium and mucosal inflammation is still poorly understood. The ENS itself is complex, with subtypes of neurons expressing 145 kd neurofilament (26%), nitric oxide synthase (NOS) (>50%), calbindin (>25%), and calretinin (>50%), with immunoreactivity to acetylcholine (26%), VIP (26%), and serotonin (<1%), among others^37^. Enteric glia can also be subdivided based on morphology and GFAP reactivity^38,39^; S100β and GFAP expressing glia were present in ENS cultures, Fig. 3(e–h). Enteric glia also impact epithelial health; their knockout or ablation leads to increased intestinal permeability, lower expression of F-Actin and ZO-1^9^, and alters the neurochemical coding of enteric neurons, decreasing choline acetyltransferase (ChAT) expression and hampering intestinal motility^40^. Enteric glia have been shown to regulate epithelial proliferation through the release of TGF-β1^41^, in agreement with our results. Increased TGF-β1 was observed in enteric cocultures compared to myofibroblast cocultures and epithelium only, Fig. 7, and increased cell density was apparent in myofibroblast cocultures compared to ENS (40% higher) and epithelium only (100% higher), Fig. 6. Further, the presence of enteric cultures altered the differentiation of intestinal stem cells, increasing the number of ChgA positive enteroendocrine cells, Fig. 6(b,e).

Myofibroblasts have previously been used to support intestinal stem cells *in vitro*^14,42^, increasing both proliferation and viability. To account for the contribution of myofibroblasts, a myofibroblast only population was also compared to complete ENS cultures, which contained neurons, glia, and myofibroblasts, Fig. 3. Cytokine production was compared for complete enteric cultures and cultures of myofibroblasts without intestinal epithelial cells, Fig. 3. In this basal state, these monocultures produced a variety of both pro- and anti- inflammatory cytokines, which help to regulate intestinal inflammation and modulate permeability^43^; however, there were no significant differences in cytokine production between the myofibroblast and complete ENS cultures, suggesting epithelial contribution stimulates differential cytokine production by these underlying cells.

The effect of primary cultures of either enteric neurons and glia or cultures of myofibroblasts on epithelial health was then determined. Culture of stem cell derived epithelium with ENS or myofibroblast cultures altered the morphology of the epithelium, Fig. 4, increasing expression of both ZO-1 and F-actin; downregulation of which is implicated in intestinal diseases such as Crohn’s, where intestinal contents flow through a leaky epithelial barrier and cause inflammation^44^. F-actin is visually increased in cultures of myofibroblasts compared to those with ENS, although this may be due to a higher density of cells, Fig. 6. Monolayers also appear more homogenous upon coculture with neurons and glia or myofibroblasts. These results agree with previous reports, where myofibroblasts and the ENS affect permeability of the intestinal epithelial barrier^15,45^ through the release of various factors, including cytokines, S-Nitrosoglutathione^9^, and vasoactive intestinal peptide (VIP)^41^, among others. Functional analyses of the epithelial monolayers demonstrated decreased epithelial permeability in epithelial cells in cocultures, as expected based on the increased expression of tight junction ZO-1, Fig. 5 and previous reports^46^. Transepithelial resistance (TEER) across the epithelia was increased and dextran flux was decreased in cocultures, signifying a less permeable monolayer. Alkaline phosphatase activity remained unchanged and was not influenced by addition of trophic cultures.

The production and release of cytokines by both myofibroblasts and glia likely impact epithelial proliferation, differentiation, and integrity due to their close proximity *in vivo*, and proinflammatory cytokine release is seen in the presentation of Crohn’s Disease and IBD^11,47–50^. Cytokines influence intestinal health though regulation of proliferation and tight junction expression, and are thus a vital signaling mechanism between the intestinal epithelium and underlying cells. For example, IL-6 is produced in response to injury and stimulates proliferation of intestinal stem cells^51^ by decreasing tight junction expression, leading to increased permeability^16^, a common symptom of inflammatory intestinal diseases. IL-6 is produced by both myofibroblasts and enteric glial cells, with IL-1β increasing release of IL-6 from enteric glia^11^. This suggests an immunomodulatory role for enteric glia in the intestinal environment. Release of IL-6 by both myofibroblasts and complete ENS cultures in this study was high. The addition of epithelium to this culture decreased the amount of IL-6 in cell culture media, possibly indicating some uptake of IL-6 by the epithelium. Other inflammatory cytokines were slightly decreased in cocultures as well, including both TNF-α, IL-1β, and IFN-γ. TNF-α release has been shown to increase permeability via downregulation of ZO-1 tight junction associated proteins^52,53^; both TNF-α and IFN-γ regulate proliferation and apoptosis of intestinal epithelial cells through Wnt/β-catenin signaling mechanisms^48^.

The addition of the epithelium stimulated the production of TGF-β1 and IL-10, but only in ENS cultures, Fig. 7. Interestingly, along with MIP-2, these were the only cytokines examined with significantly higher expression in cultures with ENS versus cultures with myofibroblasts, indicating they are likely produced by enteric neurons or glia in response to epithelial cues. Of these, only IL-10 plays an anti-inflammatory role in the gut^54^, inhibiting the production of proinflammatory cytokines; thus, glia may play an anti-inflammatory role in the gut, as their dysfunction has previously been shown to lead to inflammation^9,12^. TGF-β1 inhibits proliferation of intestinal epithelial cells in Caco-2 models, which may account for the increased number of epithelial cells in myofibroblast cocultures, Fig. 5, compared to cocultures with ENS or epithelium alone^41^. This further supports the role of various cell types surrounding intestinal crypts, including enteric cells, in regulation of both the stem cell niche and epithelial barrier function. Ongoing studies are aimed at identifying which epithelial produced cues activate the pro/anti-inflammatory cascade in the ENS.

This platform provides a coculture model for the study of enteric-epithelial crosstalk, which is easily adaptable for specific perturbations and investigation into intestinal health and disease. Initial characterization of this system has demonstrated the impact of trophic cells on epithelial stem cell differentiation and barrier integrity. The crosstalk between the ENS and epithelium remains poorly understood, and future studies utilizing this platform will determine alterations in function of both epithelium and neurons due to short chain fatty acid additions to the apical chamber, which affect both epithelial and neural function^55,56^.

## Methods

### Stem Cell Harvest and Culture

Intestinal stem cells were harvested from the duodenum of adult C57BL/6 (Charles River, MA) mice as previously described^25^. Cells were grown as organoids in 25 uL droplets of GFR Matrigel^®^ (Corning) in 24 well plates in Advanced DMEM/F12 (Gibco) containing 50% L-WRN (ATCC) conditioned media^25^, B27 and N2 supplements (Gibco), Glutamax (Gibco), N-acetyl cysteine (Sigma), HEPES (Sigma), primocin (InvivoGen), and murine epidermal growth factor (EGF, Peprotech) under standard growth conditions (37 °C, 5% CO_2_). Cells were fed every other day and were passaged every 3–5 days, or when organoids began to contact one another or darken. Organoids used in this study had previously been cryopreserved for up to 6 months in liquid nitrogen.

### ENS Harvest and Culture

Enteric cultures were harvested from the entire length of the mouse small intestines of 8–12 week male C57BL/6 mice (Taconic Farms, NY), with a protocol adapted from Smith *et al*.^57^. Briefly, the longitudinal muscle containing the myenteric plexus was stripped from the underlying circular muscle and mucosa. It was cut into approximately 1 mm^2^ sections and digested in Collagenase type II (Gibco) followed by a 0.05% trypsin/EDTA solution, for one hour and 5 minutes, respectively. Manual triteration with a reduced bore pasteur pipet was used to break segments into small clusters of cells (not single cells). Cells were seeded onto poly-D-lysine (Corning) and laminin (Gibco) coated coated coverslips, and were grown under standard conditions (5% CO_2_, 37 °C) for 7 days to allow synaptic connections to form before use in experiments. ENS Media consisted of Neurobasal A (Gibco), 2 mM L-glutamine (Sigma), 1% Antibiotic/Antimycotic (Corning), 1× B27 supplement (Gibco), 1% FBS (Corning), and 50 ng/mL murine glial derived growth factor (GDNF, Neuromics). Half of the media was replaced every other day.

### Myofibroblast Culture

Myofibroblasts were obtained from primary ENS culture by passaging and replating onto standard tissue culture flasks. Neurons and glia did not survive this transition, resulting in high purity culture of myofibroblasts as determined by broad α-smooth muscle actin expression. Myofibroblasts were cultured up to the fourth passage in Advanced DMEM containing 20% FBS, 1% Pen/Strep, and 2 mM L-glutamine. Myofibroblasts used in this study had been cryopreserved for up to 6 months in liquid nitrogen.

### Transwell and Myofibroblast Seeding

Transwell inserts (0.4 um pores, PET, Corning) were coated with 0.2 mg/mL rat tail collagen type 1 (Corning) prior to seeding of intestinal stem cells. Collagen was added to plasma treated transwells for 15 minutes, and excess solution was removed. Transwells were dried in a cell culture hood for 2 hours. Cryopreserved myofibroblasts were seeded at 50,000 cells per cm^2^ in tissue culture 24 well plates. Myofibroblasts were allowed to attach to the plates for 2 to 3 hours before the addition of transwells and seeding of intestinal stem cells. Intestinal stem cells were removed from Matrigel^®^ and dissociated with EDTA in HBSS followed by 5 minutes of 0.05% Trypsin in HBSS. Cells were triturated into a single cell solution. Approximately 250,000 cells were seeded per transwell (SA: 0.33 cm^2^) in intestinal stem cell culture media supplemented with 10 μM rho kinase inhibitor (Tocris Y-27632) to prevent apoptosis. Media was replaced after 24 hours with experimental media to promote differentiation, which contained 10% FBS and 10% WRN conditioned media in Advanced DMEM with 1% penicillin – streptomycin. Cells were grown for 3 days before analysis.

### Monolayer Health Assays

Transepithelial resistance (TEER) was measured on days 1 and 3 with an epithelial voltmeter (EVOM2, World Precision Instruments). Media was replaced 30 minutes prior to measurements. Readings were done in triplicate and averaged for each well. Alkaline phosphatase activity was measured with a SensoLyte FDP Alkaline Phosphatase Assay Kit from individual transwells according to manufacturers instructions. Apical to basolateral flux through epithelial monolayers was determined by addition of FITC-coordinated dextran (Sigma) to cell culture media (2.2 mg/ml, Sigma) in the apical transwell chamber. Basolateral media was sampled every 30 min for 2 hours to determine dextran flux. FITC-dextran was quantified on a fluorescent plate reader. Analyses were done in triplicate for 3 separate experiments (alkaline phosphatase and dextran flux) or 6 experiments (TEER).

### Immunostaining and Imaging

Cells were fixed with 4% paraformaldehyde (Sigma) for 30 minutes, permeabilized with 0.1% triton X-100 (Sigma) for 15 minutes, and blocked in 2.5% goat serum (Sigma) for one hour. Primary antibodies were applied in goat serum for one hour. Cells were rinsed thrice for 15 minutes with PBS after removal of PFA, triton X-100, primary, and secondary antibodies. Primary antibodies used for enteric neuron and myofibroblast cultures were β-III tubulin (Invitrogen, 1:500 v/v%), neurofilament medium (Invitrogen, 1:500), calretinin (Invitrogen, 1:500), vasoactive intestinal peptide, (VIP, Abcam, 1:250), choline acetyltransferase (ChAT, Invitrogen, 1:250), synapsin (Abcam, 1:500), glial fibrillatory acid protein (GFAP, Invitrogen, 1:300), α-SMA (Invitrogen, 1:200), and S100β (Dako, 1:400). For intestinal stem cell cultures, primary Mucin-2 (Invitrogen, 1:300) was used. Secondary Alexa Fluor 488 and 546 were applied for one hour in blocking agent at 1:1000 v/v%. Alexafluor 647 Phalloidin was applied with secondaries at a 1:1000 dilution. Transwell membranes were removed and mounted on slides with Prolong Gold Mount with DAPI Antifade (Invitrogen). A coverslip was applied over the membrane and sealed with clear nail polish. Mounted transwells and coverslips were imaged with 10×, 20×, and 40× objectives on a Zeiss Axio Observer fluorescent microscope (Carl Zeiss), and average image intensities were quantified with Zeiss Zen software.

### Cytokine Analyses

Multiplex ELISA was used to determine soluble cytokine concentrations from myofibroblasts and ENS in both apical and basolateral chambers of cocultures. Media was collected after 48 hours of incubation on day 3 before further functional analyses or fixation. A Bio-Plex Pro^™^ Mouse Cytokine Th17 Panel A 6-Plex (Bio-Rad Laboratories, Inc) was used to quantify production of IFN-γ, IL-1β, IL-6, IL-10, IL-17A, and TNF-α. Singleplex ELISAs were performed for MIP-2 and TGF-β1 (ProcartaPlex, Invitrogen). TGF-β1 was activated immediately before analysis via a 10 minute incubation with 1N Hydrochloric Acid (Fisher Sci) and subsequent neutralization with 1.2N Sodium Hydroxide/0.5M HEPES. All cytokine assays were analyzed on a Luminex 200 system (Bio-Rad Laboratories, Inc) according to manufacturer instructions.

### Animal Care and Use

The institutional tissue isolation protocol was approved by Northeastern University’s Institutional Animal Care and Use Committee (IACUC). All procedures were performed according to relevant guidelines and regulations.

### Statistics

All statistical analyses were performed in IBM SPSS. ANOVA or a two-tailed t-test were used to determine significance, which was considered p < 0.05 for all results unless otherwise noted.
